# Distinguishing functions of trypanosomatid protein kinases

**DOI:** 10.1016/j.pt.2022.08.009

**Published:** 2022-09-05

**Authors:** Mathieu Cayla, Y. Romina Nievas, Keith R. Matthews, Jeremy C. Mottram

**Affiliations:** 1 Institute for Immunology and Infection Research, School of Biological Sciences, University of Edinburgh, Edinburgh, UK; 2 York Biomedical Research Institute, Department of Biology, University of York, York, UK

## Abstract

Trypanosomatid parasitic protozoa are divergent from opisthokont models and have evolved unique mechanisms to regulate their complex life cycles and to adapt to a range of hosts. Understanding how these organisms respond, adapt, and persist in their different hosts could reveal optimal drug-control strategies. Protein kinases are fundamental to many biological processes such as cell cycle control, adaptation to stress, and cellular differentiation. Therefore, we have focused this review on the features and functions of protein kinases that distinguish trypanosomatid kinomes from other eukaryotes. We describe the latest research, highlighting similarities and differences between two groups of trypanosomatid parasites, *Leishmania* and African trypanosomes.

## Investigating the kinomes of trypanosomatids

**Figure 1:**
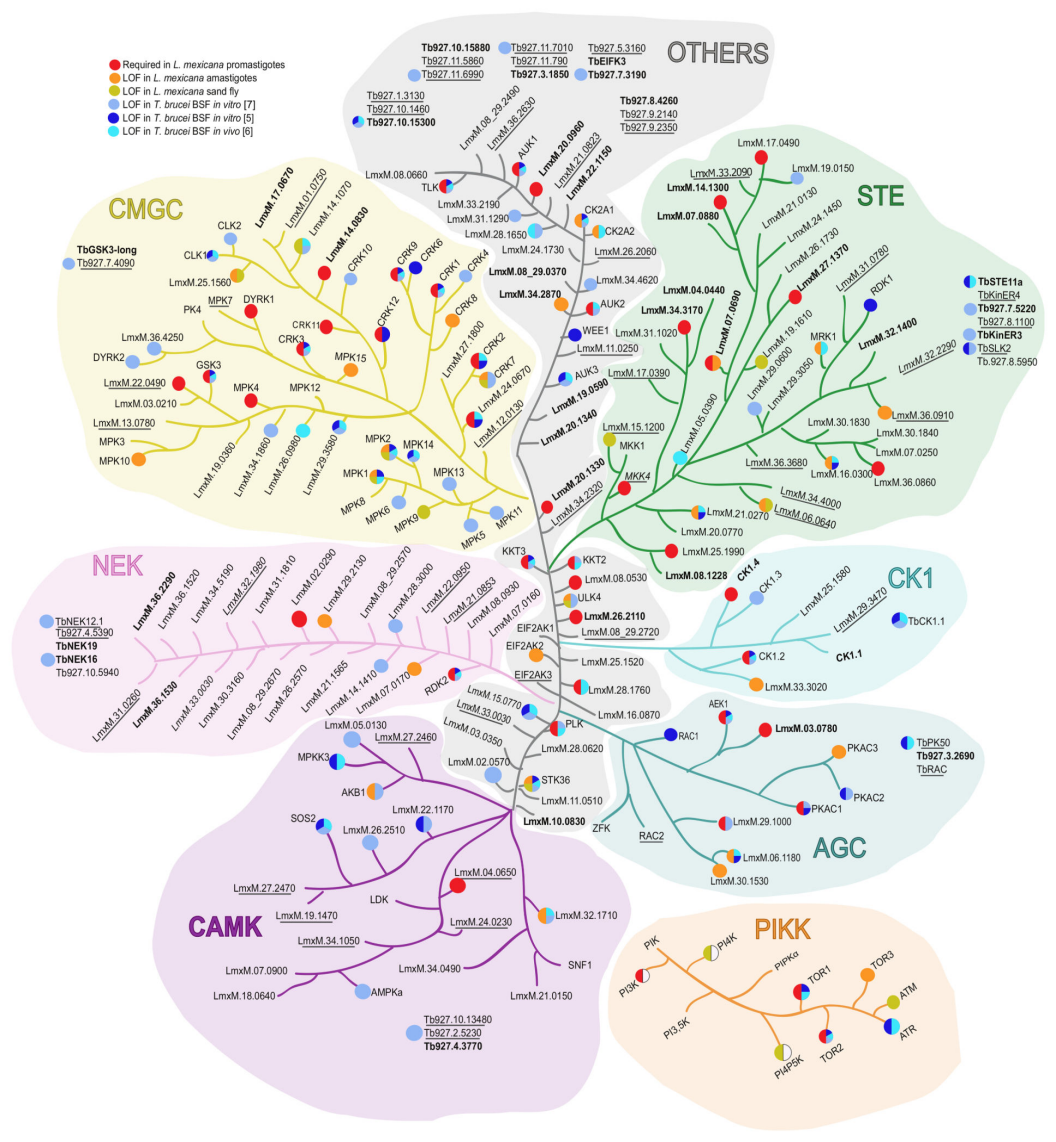
*Leishmania mexicana* and *Trypanosoma brucei* kinomes. Representation of eukaryotic protein kinases (ePKs) and the subfamily of phosphatidylinositol 3′ kinase-related kinases (PIKKs) of atypical protein kinases (aPKs). Each family is separated by colours defined by primary protein sequence conservation. Image for illustrative purpose only with no phylogenetic significance. The colours filling the dots represent as follows: red, required in *L. mexicana* promastigotes; orange, loss of fitness (LOF) in *L. mexicana* amastigotes; green, LOF for sand fly infection; dark blue, LOF in bloodstream form (BSF) *T. brucei in vitro* [5]; turquoise, LOF in BSF *T. brucei in vivo* [6]; light blue, LOF in BSF *T. brucei in vitro* [7]. The protein kinases unique either to *Leishmaniinae* or to *Trypanosomatinae* are indicated in bold. Underlined *L. mexicana* protein kinases are absent in *T. brucei* only and underlined *T. brucei* protein kinases are absent in *L. mexicana* only.

*Trypanosoma* and *Leishmania* protozoan parasites are clinically important human pathogens that cause African sleeping sickness, Chagas’ disease, and distinct forms of leishmaniasis worldwide, as well as nagana, dourine, and leishmaniasis in mammalian animals. Their life cycles require proliferation and a series of cellular differentiation steps, generating parasite forms adapted to their mammalian and haematophagous insect hosts. As in other eukaryotes, such processes are regulated by phosphorylation-mediated signal-transduction events (Box 1) controlled by the antagonist action of protein phosphatases and **protein kinases** (see Glossary). The protein kinase superfamily is composed of **atypical protein kinases (aPKs)** and **eukaryotic protein kinases (ePKs)**. The aPKs comprise 28 and 20 members in *Leishmania mexicana* and *Trypanosoma brucei*, respectively. ePKs are classified into two superfamilies: **protein serine/threonine kinases**, which are ubiquitous to all eukaryotes; and **protein tyrosine kinases** that are absent in most unicellular eukaryotes, including trypanosomatids. Tyrosine phosphorylation is nonetheless documented in these parasites [1] and is carried out by dual-specificity kinases. Genomic searches for trypanosomatid kinomes identified 193 and 157 ePKs in *L. mexicana* and *T. brucei*, respectively [2–6], with 140 ePKs orthologues shared between *L. mexicana* and *T. brucei*, as annotated in TriTrypDB (Figure 1). These parasites also encode species-specific protein kinases as exemplified in *L. mexicana*, which encodes 24 protein kinases unique to the *Leishmaniinae* and another 34 that are absent from *T. brucei*, whereas *T. brucei* encodes 13 protein kinases that are unique to the *Trypanosomatinae* and 21 that are absent from the *L. mexicana* kinome. The trypanosomatids’ kinomes are about one-third the size of that in humans, and about twice the size of that in the malaria parasite *Plasmodium falciparum*, which is intriguing considering that these parasites have similarly complex life cycles.

In a genome-wide **RNA interference target-sequencing (RIT-seq)** screen, Alsford *et al*. identified protein kinases required for *T. brucei* bloodstream form fitness after RNAi knockdown *in vitro* (40 protein kinases at day 3 post-induction and 56 at day 6) [7]. Later, Jones and colleagues generated a kinome-focused RNAi library targeting 176 individual proteins in *T. brucei* bloodstream forms and identified 42 protein kinases required for parasite proliferation in culture [5]. Only half of the identified protein kinases were shared between both studies, and the authors attribute the variance to the use of different *T. brucei* strains, RNA constructs, and methods for assessing cell growth. A complementary RIT-seq analysis of the pooled kinome-focused library after mouse infection over 72 h identified a further nine protein kinases that appear to be more important for survival in the mammalian host than in culture [6]. The recent development of CRISPR/Cas9 editing has enabled the study of the *L. mexicana* kinome, allowing comparison of protein kinase functions between trypanosomatid parasites. This kinome-wide gene deletion **bar-seq, loss-of-fitness (LOF)** screen highlighted that 35% of *L. mexicana* protein kinases are required for survival in the three different life stages assessed (43 in culture promastigotes and 29 in amastigote *in vivo* and *in vitro*, and 15 in forms found in the sand fly insect vector) [2]. Comparison of the LOF data for each protein kinase in *Leishmania* and bloodstream-form African trypanosomes revealed little correlation between essential stage-specific protein kinases (Figure 1 and Table S1 in the supplemental information online). For example, 45% of the kinome is required for the survival of *T. brucei* bloodstream forms, and only 19 out of 27 of the ‘essential’ *L. mexicana* protein kinases that have orthologues in *T. brucei* (70%) are also found to be required for the bloodstream form. The correlation between the *T. brucei* and *L. mexicana* orthologous protein kinases exhibiting LOF in the mammalian stage is lower than between different life stages of each parasite (55%). However, it remains to be determined if the function of the protein kinases required for the *Leishmania* promastigote stage is the same in the amastigote stage, for example, and further conditional gene-deletion experiments will be necessary to answer this. These studies have also revealed that only 40% of ‘unique’ protein kinases ‘in *T. brucei* and 25% in *L. mexicana* are required at any one life stage.

Trypanosomatid kinomes possess homologues of five major ePKs groups: **CMGC, AGC, CAMK, STE**, and **CK1**. The remaining ePKs that do not fall into these groups are categorized as ‘Other’. CAMK and AGC groups are poorly represented in trypanosomatid kinomes, whereas CMGC, STE, and Other-**NEK** families are expanded [3]. The never-in-mitosis gene A (NIMA)-related kinase (NEK) family promotes cell cycle events in eukaryotes. Despite being expanded in *Leishmania*, NEK family members are largely dispensable in the promastigote stage as gene deletion mutants could be generated for 18/20 members [2]. In all groups, a similar number of protein kinases are required in each parasite, except for the CAMK group, which have more protein kinases with a LOF phenotype in *T. brucei* bloodstream (around 50%) compared to 15% in the different *Leishmania* life stages. Trypanosomatid cells have a highly polarized morphology that changes during their life cycle; thus, the expansion of the CMGC group may ensure stringent control of the replication and segregation of organelles during both their cell cycle and their life cycle. In this regard, most of the CMGC kinases that are required for survival are well studied regulators of the cell cycle, such as CDC2-related protein kinases (CRKs), CDC-like protein kinases (CLKs) or GSK3 (Box 2). However, other CMGC kinases identified in the *L. mexicana* kinome-wide screen had a LOF in the amastigote (i.e., CRK7, CRK8, MPK10) or the sand fly stages (MPK9, LmxM.14.1070), suggesting that they may be stage-specific regulators. In summary, 60% of CMGC protein kinases are required for survival of the bloodstream form of *T. brucei*, whereas 43% are required for *L. mexicana*. The STE group includes Ste7/MAP2K, Ste11/MAP3K, and Ste20/MAP4K that function as upstream regulators of MAP kinases. This is the second largest protein kinase group in trypanosomatids, comprising mainly orthologues of STE11 and STE7; STE20 orthologues are rare [3]. Little is known of their function but ~40% have LOF phenotypes in both parasites [2,5,8,9].

Overall, these studies have revealed that the trypanosomatids contain orthologues of many conserved eukaryotic protein kinases with similar anticipated functions to those in mammalian cells. However, other protein kinases are likely to have parasite-specific functions, such as coordinating the duplication of specific organelles or differentiation events. This review provides the current state of knowledge on the role of protein kinases in trypanosomatids, focusing on the unique and unusual features that reflect the complexity of their biology.

## Protein kinases and the cell cycle

Trypanosomatids are evolutionarily divergent eukaryotes and exhibit unique features in their cell cycle, many of which will be regulated by protein kinases. This includes their unique apparatus for chromosome segregation and the regulatory processes governing the highly orchestrated duplication and segregation of single-copy organelles. Conserved functions for protein kinases in cell cycle control are discussed in Box 2.

### Chromosome segregation

During mitosis, the macromolecular structure providing the major attachment point for spindle microtubules is the kinetochore, which assembles on the centromeres of chromosomes. Strikingly, trypanosomatids lack classical homologues of most of the kinetochore components that are ubiquitously conserved among yeasts, plants, and mammals. Instead, the inner trypanosomatid kinetochore is composed of at least 26 distinct and essential proteins (KKT1-26) that include four protein kinases, CLK1 (KKT10), CLK2 (KKT19), KKT2, and KKT3 [10,11]. CLK1 and CLK2 have overlapping functions and are required for the phosphorylation of KKT2, KKT4, and KKT7 [10–12]. These kinases exhibit a dynamic localisation at kinetochores during metaphase and disappear at the onset of anaphase, events requiring CLK1 to interact with KKT7 and the KKT8 complex [13]. A specific amidobenzimidazole inhibitor of CLK1 (AB1) was identified from using a phenotypic screen against bloodstream forms of *T. brucei* [12]. CLK1 was irreversibly inhibited by AB1 by forming a covalent bond with Cys215 in the ATP-binding pocket, a residue that is absent from human CLK1. Importantly, chemical inhibition of CLK1 by AB1 led to chromosome mis-segregation and cell cycle arrest, leading to cell death of *T. brucei*. AB1 also exhibited activity against *T. cruzi, L. mexicana*, and *Leishmania donovani*, suggesting that CLK1 is a pan-trypanosomatid drug target [12]. The peculiar TbECK1, which shares features of **mitogen-activated protein kinases (MAPKs)** and cyclin-dependent protein kinases (CDKs), may also be involved in chromosome segregation as aberrant nuclear and kinetoplast configurations and DNA content were observed upon expression of a truncated protein lacking its regulatory C-terminal extension [14].

### Cytokinesis and organelle segregation

Successful cell division of trypanosomatid parasites requires that they accurately replicate and segregate several single-copy organelles to each daughter. This includes their flagellum and kinetoplast, a large concentration of mitochondrial DNA segregated through tethering to the cytoskeleton through the basal body [15], a structure related to centrioles of the eukaryotic nuclear spindle pole. Several protein kinases and protein kinase families have been found to contribute to these events. Polo-like kinases (PLKs) play an important role in a variety of mitotic events in mammalian cells, ranging from centriole separation and chromosome condensation to abscission. To fulfil these roles, PLK homologues have been observed at different cellular locations as the cell cycle progresses, with PLK at the centrosome, the spindle poles, and the midbody. In *T. brucei*, the single PLK homologue is essential for cytokinesis and is necessary for the correct duplication of the centrin-containing bilobe, a cytoskeletal structure that serves as a scaffold for Golgi duplication [16]. Unlike all the other nuclear eukaryotic PLKs that control both mitosis and cytokinesis, PLK localizes to the basal body and only regulates cytokinesis [17]. Indeed, PLK localizes to the basal body, then successively locates to a series of cytoskeletal structures that regulate the position and attachment of the flagellum to the cell body by association with a specialized set of microtubules, known as the microtubule quartet [18]. This observed change of localisation and abundance is controlled by interaction with a Cullin-RING ubiquitin ligase complex that degrades PLK in the basal body and the bilobe after the G1/S cell cycle transition. This degradation promotes bilobe duplication, basal body separation, and flagellum–cell body adhesion [19]. Interestingly, amongst the 23 protein kinases that are located to the basal body in *L. mexicana* only two are essential for parasite survival, one of which is PLK [2]. TOEFAZ1, an essential trypanosomatid-specific component of the *T. brucei* cytokinetic machinery, is reported to be phosphorylated by PLK to allow cytokinesis initiation or the initiation of cleavage furrow ingression [20,21]. Similarly, PLK phosphorylates SPBB1, a basal body protein, to promote basal body segregation and the initiation of the flagellar attachment zone (FAZ) filament to allow adhesion of the flagellum and the initiation of cytokinesis [22]. Finally, PLK acts antagonistically with the phosphatase KPP1 to regulate flagellum inheritance during parasite cytokinesis [21]. Specifically, KPP1 dephosphorylates PLK, reducing its phosphotransferase activity, thus reducing the hyper phosphorylation of TbCentrin2, a step required for the completion of the hook complex duplication, enabling correct flagellum inheritance during cell division [23,24].

The mammalian NIMA-related kinase 2 (NEK2) has important cell cycle functions related to centriole integrity and division. In *T. brucei*, NEK-related kinase NRKC is located on mature and immature basal bodies and is implicated in basal body segregation during cytokinesis [25]. NEK12.1, one of only two *T. brucei* ePKs to possess a small gatekeeper residue in its ATP binding pocket, and NEK12.2 (repressor of differentiation kinase RDK2) have also been demonstrated to regulate kinetoplast division and cytokinesis [5]. The depletion of both genes resulted in LOF both in culture and in a mouse model. As well as cell cycle control, NEK12.2 and several other NEK family members are also involved in cell differentiation events, as detailed in subsequent text.

### Gene transcription

In trypanosomatids, transcription occurs polycistronically and individual mRNAs are processed from a precursor by trans-splicing of a **spliced leader (SL)** sequence at the 5′ end and polyadenylation at the 3′ end. While several CRKs are implicated in cell cycle regulation (Box 1), CRK9 is essential for the first step of splicing. CRK9 depletion triggers a reduction of mature mRNAs and an increase in unspliced pre-mRNAs, accompanied by a reduction of the phosphorylation of the RNA polymerase (pol) II subunit RBP1 [26,27]. CRK9 requires L-type cyclin (CYC12) and CRK9-associated protein (CRK9AP) to form a tripartite complex resulting in autophosphorylation and activation of CRK9 [28]. Protein kinase activity is also required for the transcription of the multicopy SL RNA gene locus that depends upon the snRNA-activating protein complex (SNAPc) and the TATA-binding protein TBP-related factor 4 (TRF4). Upon endoplasmic reticulum (ER) stress, the homologue of eukaryotic initiation factor 2 (eIF2) kinase TbeIF2K3 (alias PK3) in *T. brucei*, migrates from the ER membrane to the nucleus, and phosphorylates TRF4. This phosphorylation on S35 leads to TRF4 release from the defined polymerase II promoter, resulting in SL silencing and blockage of RNA transcription [29,30].

## Protein kinases with distinct roles in *Leishmania* and trypanosomes

The complex life cycles of trypanosomatid parasites involve environmental fluctuations accompanied by extensive metabolic, morphological, gene, and protein expression changes. Researchers have exploited different methods to explore the regulatory control of these events, revealing both shared underlying controls and parasite-specific mechanisms.

**Figure 2:**
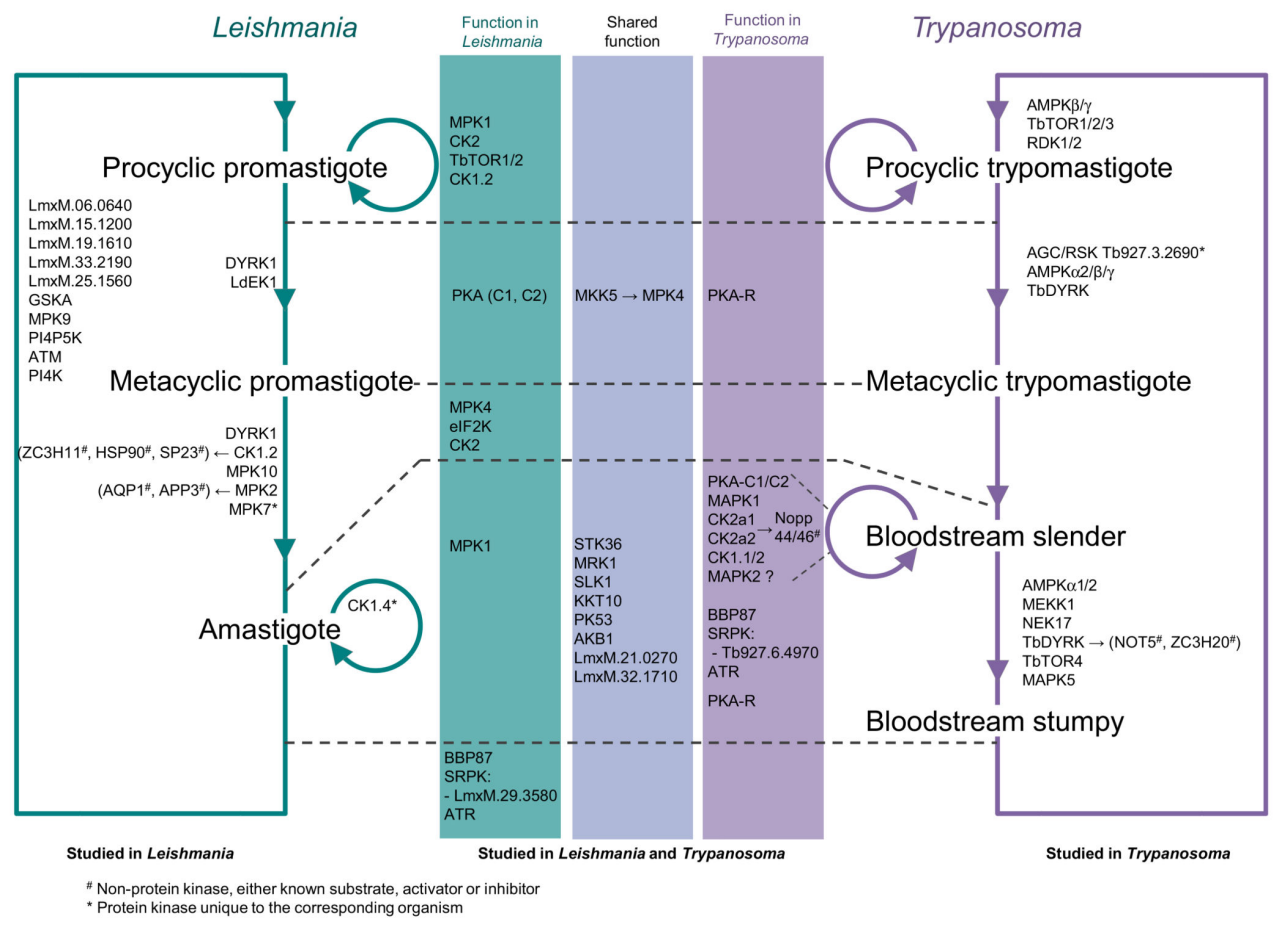
Graphic representation of protein kinases individually studied and involved in cellular differentiation of *Leishmania* and *Trypanosoma*. The position of each protein kinase indicates in which developmental stage they are implicated. Protein kinases studied in both organisms are separated in the central area, in regard to their function being shared in both organisms (light purple colour) or specific for either *Leishmania* (teal colour) or *Trypanosoma* (dark purple colour). Protein kinases included within the life cycles have been studied only in the corresponding organism. ^#^Nonprotein kinase, either substrate, activator, or inhibitor of protein kinase; *Protein kinase unique to the corresponding organism.

The comparison of the kinome-wide screens investigating protein kinase functions at different life stages of trypanosomatid parasites revealed that a number of protein kinases generated a LOF phenotype in both *T. brucei* mammalian bloodstream forms and during amastigote formation in *Leishmania* (MPK1, CK2a1, CK2a2, STK36, MRK1, SLK1, KKT10, PK53, AKB1, LmxM.21.0270, and LmxM.32.1710) or during the establishment of fly infection (BBP87, SRPK: Tb927.6.4970, and the atypical ATR phosphatidylinositol 3-related kinase) [2,5,6]. To date only a few protein kinases have been studied individually in both trypanosomatid species for their respective function in cellular differentiation and host survival and adaptation. In the following paragraph, and Figure 2, we provide examples of these protein kinases.

Protein kinase A (PKA) has been discovered in most eukaryotes, except plants, and is composed of regulatory PKAR and catalytic PKAC subunits either as heterodimer or heterotetramer. Upon binding of the second messenger cAMP, PKAC is released from PKAR and activated. The *T. brucei* genome encodes three orthologues of PKAC, and one PKAR. Both PKAC1 and PKAC2 are required for optimal parasite growth [5,7]. The PKAR subunit has been identified as a potential component of the trypanosome quorum-sensing differentiation pathway in a genome-wide RNAi screen in response to cell permeable cAMP analogues [9]. Additionally, PKAR is required for *in vitro* metacyclogenesis following overexpression of the RNA regulator RBP6 [31]. Homology modelling and crystallisation of PKAR suggested that this subunit is unable to form a homodimer [32] and confirmed that key amino acids in both CNB domains (required for PKAC binding) together with a unique C-terminal αD helix account for the interesting cAMP-independent activation mechanism of PKA [33,34]. In *Leishmania*, PKA expression and activity increases upon starvation, leading to an increase in autophagy and metacyclogenesis [35–37]. In contrast to *T. brucei, Leishmania* PKA is inhibited by the phosphodiesterase PDEA (that degrades cAMP) and activated by adenylate cyclase (involved in cAMP synthesis) [37] suggesting a role for cAMP in the PKA pathway. *L. donovani* PDEA is reported to interact with PKAC1 and PKAC2, resulting in protein kinase activity and an increase in the phosphodiesterase activity through PKA-mediated phosphorylation [38]. Taken together, these observations are not easily reconciled and the role of cAMP in PKA function in trypanosomatid organisms needs to be resolved.

MPK4 and MPK1 have also been studied for their function during differentiation in *Leishmania* and *Trypanosoma. Leishmania* MPK4 appears to be involved in pH sensing during metacyclogenesis [39]. MPK4 is activated by the MAP kinase MKK5 by direct phosphorylation on the activation loop residues T190 and Y192 [40]. Another *Leishmania* MAPK, MPK1, is required for differentiation and/or survival in both the insect vector and mammalian host [2]. In *L. donovani* [41] it is associated with HSP70/90 of the foldosome complex [42,43] and acts in the negative regulation of P-glycoprotein-mediated efflux pump activity, leading to antimony sensitivity. These observations were supported by the analysis of antimony-resistant *Leishmania* virulent field strains, which exhibit a reduction in MPK1 expression [44].

Membrane-bound eIF2a protein kinases have been identified in the flagellar pocket of *T. brucei* (eIF2K), suggesting a relevance in environmental sensing or nutrient transport, whereas in *L. infantum* eIF2K is a transmembrane kinase located on the endoplasmic reticulum. Its kinase activity increases through autophosphorylation in response to different stresses. *In vitro*, the active protein kinase phosphorylates the translation initiation factor 2-alpha subunit (eIF2a) at threonine 166 [45], and *in vivo* is essential for the differentiation of *Leishmania* to amastigote forms [2,46].

## Protein kinases implicated in differentiation events

### *T. brucei* long slender to short stumpy bloodstream trypomastigote development

For *T. brucei*, the quorum sensing (QS)-mediated development between proliferative slender forms and transmissible stumpy forms was analysed through a genome-wide RNAi screen. This screen was performed in a laboratory-adapted monomorphic strain and exploited cell-permeable analogues of cAMP or AMP to mimic the QS signal. This identified a number of protein kinases driving stumpy formation, including a 5’ adenosine monophosphate-activated protein kinase homologue AMPKα2, a MEK kinase MEKK1, the NEK17 kinases, and a DYRK/YAK kinase, DYRK [9]. Each of these protein kinases are involved in the stumpy form developmental pathway that seems to be organised in a nonlinear hierarchy [47]. DYRK shares characteristics with the eukaryotic DYRK2 family while having unique characteristics such as mutations of key residues leading to a possible preactivated state, a so-called ‘DFS-in’ state, and three insertions within the kinase domain. Interestingly, this protein kinase acts on both positive and negative regulators of stumpy formation by phosphorylating and activating ‘stumpy-inducer’ proteins such as the zinc finger protein ZC3H20 and phosphorylating and inhibiting ‘slender-retainer’ proteins such as the component of the CAF1/NOT deadenylation complex NOT5 [48].

### *T. brucei* procyclic trypomastigote formation

Once inside the tsetse fly, stumpy forms differentiate to replicative procyclic forms, which eventually differentiate into the mammal infective metacyclic forms. A MKK1 null mutant in the procyclic stage leads to parasites incapable of colonising the salivary glands of tsetse flies [49]. A kinome-wide RNAi screen identified two kinases, RDK1 and RDK2, whose depletion promoted blood-stream to procyclic form differentiation. It was shown that RDK1, a STE-like protein kinase, acts together with a phosphatase cascade comprising PTP1 and TbPIP39 to block uncontrolled differentiation, while RDK2 (NEK12.2) depletion triggers spontaneous differentiation of blood-stream to procyclic forms [5]. Additionally, NRKA and NRKB mRNAs are enriched in stumpy forms and have been implicated in procyclic differentiation, likely operating downstream of the PTP1 and PIP39 phosphatases [50].

### *T. brucei* metacyclic trypomastigote formation

Using the expression of one of the major metacyclic VSGs, mVSG397, as a read out for metacyclogenesis, Toh *et al*. have determined that the AGC/RSK, unique to trypanosomes, is involved in metacyclogenesis [31]. In this analysis they also identified several other protein kinases involved in metacyclogenesis that have been previously implicated in QS-dependent differentiation, such as AMPKα2, AMPKβ, and AMPKγ, PKAR and DYRK [31]. Additionally, RDK2 was also found to be upregulated in metacyclic cells evidenced by proteomics analysis [51]. The life cycle of *T. brucei* is completed when metacyclic trypomastigotes are transmitted to a new host upon a blood meal and differentiate to the slender form, but the protein kinases involved in this stage remain to be elucidated due to the technical challenges of studying this transition.

### *Leishmania* differentiation within the mammalian host

*Leishmania* differentiation in the mammal involves host cell occupancy. Thus, the elongated-flagellated and cell-cycle-arrested metacyclic promastigote differentiates to an intracellular non-motile and replicative amastigote form within the parasitophorous vacuole of host macrophages. In *L. infantum*, a member of the DYRK family, DYRK1, that localises mainly in the flagellar pocket and on endosomes during logarithmic growth, becomes recruited to the mitochondrion during late stationary phase. This molecule is required to sustain stationary phase, metacyclogenesis. and macrophage infection [52]. Notably, however, a DYRK1 null mutant was not achieved in *L. mexicana* [2], suggesting different mechanisms in the two species.

Casein kinases CK1 are also essential for amastigote intracellular survival since specific inhibitors, besides blocking promastigote growth, reduce axenic amastigote viability and decrease the number of intracellular amastigotes in infected macrophages [53,54]. Depletion of CK1.2 resulted in an increase in postmitotic and abnormal cells, suggesting direct or indirect effects on kineto-plast division and cytokinesis [5]. CK1.2 has, as substrates, ZC3H11 [55], HSP90 [56], SP23, and the related P23 cochaperone [57]. It is also released into the extracellular environment to phosphorylate host proteins, such as the complement factor C3a or the interferon receptor IFNAR1, to modulate the host immune response [58]. The overexpression of CK1.4 in *L. donovani* induces parasites to grow at higher density and results in higher macrophage infection [59], suggesting that a specific role evolved in *Leishmania* for intracellular parasite survival and proliferation.

Amongst MAPKs, MPK10 is required for differentiation and/or survival in the mammalian host [2]. It is an interesting protein kinase because the crystal structure [60] and a structure/function analysis [61] revealed a constitutively active state, with the ‘DFG-in’ conformation observed in activated kinases, and an autoinhibitory C-terminal domain negatively regulated by phosphorylation during amastigote differentiation [61,62]. Like MPK10, *Leishmania* MPK2 is essential for differentiation and survival in the host [2]. MPK2 modulates intramacrophage amastigote survival by phosphorylation of the aquaglyceroporin 1 (AQP1) and an amino acid transporter (APP3), with both substrates respectively involved in drug resistance and the arginine depletion response [63,64]. The phosphorylation of AQP1 on T197 reduces its turnover and activity, modifies its localisation from the flagellum to the cell surface, and leads to an increase of uptake of antimony [Sb(III)]) [63]. MPK7 overexpression inhibits intracellular growth of the *L. donovani* amastigote stage [65], and the protein kinase activity is low in logarithmic promastigotes but increases in axenic amastigotes [62]. The deletion of MPK7 in *L. mexicana* had no LOF phenotype during promastigote-to-amastigote differentiation in Baker *et al*. [2].

### *Leishmania* development within the sand fly

Development of *Leishmania* in the sand fly is far more complex than in *in vitro* cultures and involves sequential differentiation from procyclic promastigote to the nectomonad form and then to the leptomonad form. However, because of the clear challenges of laboratory work with the sand fly, most of the information collected to date has been derived from procyclic promastigotes grown *in vitro*. For example, eK1 (LdBPK_110060.1), a *L. donovani* GCN2-like eIF2α kinase, has been found to be activated in response to starvation stress, and phosphorylates eIF2a leading to G1 arrest and metacyclogenesis [66]. Two other protein kinases unique to *Leishmania*, LmxM.06.0640 and LmxM.15.1200, had LOF phenotypes for the establishment of infection in sand fly as gene deletion mutants. Additionally, seven protein kinases have been reported to generate a LOF phenotype specifically in the sand fly (Table S1). This suggests that *Leishmania* exhibits unique functional adaptions for sand fly colonisation [2].

## Concluding remarks

The detailed dissection performed over the years of the signalling pathways mediating trypanosomatid-specific functions have revealed fascinating insight into the parasites’ complex biology. The recent development of high -throughput genetic screens and CRISPR technology has greatly facilitated the study of protein kinases, allowing comparative analysis between species. However, our analysis has highlighted the absence of systematic and comparative functional analysis of orthologous protein kinases in trypanosomes and *Leishmania*, which therefore precludes the assignment of parasite-specific or shared biological activities for the respective molecules. In addition, we have observed that, despite a general good phenotype conservation, the genetic modification of several protein kinases can lead to different phenotypes between independent studies. These differences may be dependent on the different strains, methodologies, and quality and sensitivity of the assays used, and highlight that normalisation of protocols would benefit the research community. Nevertheless, unique features of trypanosomatid kinomes suggest that the development of innovative interventions are possible to specifically target protein kinases in these pathogens. Protein kinases are established targets for the development of treatment strategies in eukaryotes, and proof-of-principle data have been obtained under laboratory conditions, demonstrating that trypanosomatid protein kinases are suitable targets, as reviewed by Field *et al*. [67]. Further discovery research is therefore required on the function of kinomes in these organisms, as well as more translational research to develop new broad multispecies control strategies (see Outstanding questions).

## Supplementary Material

**Supplemental information**

Supplemental information associated with this article can be found online at https://doi.org/10.1016/j.pt.2022.08.009.

Supplementary Material

## Box 1 Phosphorylation in trypanosomatids

Phosphorylation is a highly abundant and dynamic post-translation modification in trypanosomatids. Analysis of the phosphoproteomes of *T. brucei* bloodstream and procyclic forms identified 8275 differentially phosphorylated sites in 2233 proteins. Several protein kinases showed differential phosphorylation in the two forms analysed, including members of the MAPK signalling cascade (STE11-STE7-MAPK) together with proteins implicated in developmental events, suggesting a phosphorylation-specific regulatory role in each life cycle stage [1]. Furthermore, quantitative phosphoproteomics in synchronised *T. brucei* PCF identified 917 cell-cycle-regulated phosphorylation sites on 586 proteins. Peaks in phosphorylation abundance occurred mostly during S-phase and mitosis with two potential inhibitory sites on CRK3 (T33, Y34) and two sites on PLK (S388 and T469) exhibiting increased phosphorylation at this cell cycle stage. Consistent with a role in cytokinesis, MAPK6 and RCK showed a peak in phospho-site abundance at the early G2-M phase only (pY176 and pS344). Changes in phosphorylation during the cell cycle were identified also for kinetochore proteins, in RNA-binding proteins and in components of the translation machinery [68]. In *L. donovani*, a phospho-proteome analysis during the time course of promastigote to axenic amastigote differentiation identified peaks in phosphorylation abundance early in the differentiation signal response and during the morphological transition stages. In contrast, dephosphorylation was observed during parasite movement cessation and amastigote maturation. Several protein kinases were phosphorylated during differentiation together with proteins involved in translation and heat shock [69]. Indeed, HSP90 and HSP70 family members are proteins phosphorylated specifically in amastigote forms [70], suggesting a relationship between the adaptation to developmental programmes and stress-response processes in *Leishmania*. In *T. brucei*, a phosphoproteomic analysis of heat-shock-stimulated cells identified 193 sites in 148 proteins to be differentially phosphorylated. Several key regulators of the heat-shock response were heavily phosphorylated upon treatment, including the RNA helicase DHH1 the poly(A) binding protein PABP2 and the ZC3H11-MKT1 complex. Differentially phosphorylated protein kinases included proteins required for the survival of the bloodstream form parasite such as CK1.2, GSK3, and PLK among others [71]. In *L. mexicana*, a recent study combined *in vivo* protein crosslinked proximity biotinylation (XL-BioID) and phosphoproteomics to follow the spatial dynamics of the kinetochore assembly during the cell cycle. The method allowed the identification of sites with a specific decrease in phosphorylation but not in protein abundance, suggesting that phosphorylation of kinetochore proteins could be regulating kinetochore function. Combining proximity phosphoproteomics with chemical inhibition is a potential approach for discovery of *in vivo* protein kinase signalling pathways [11].

## Box 2 Protein kinases functioning as conserved cell division regulators

Progression through the cell cycle in trypanosomatids is regulated by cyclin-dependent kinases (CDKs), as occurs in all eukaryotes examined. Six from the 11 identified CDKs (named cdc2-related kinases, CRKs) in *T. brucei* [5] and 7 in *Leishmania* [2] are necessary for proliferation in *L. mexicana* promastigotes (CRK1-3, 9, 11-12) [2] and *T. brucei* bloodstream forms (CRK1-3, 6, 9, 12) [5,6]. *T. brucei* CRK1 is involved in regulating the G1/S transition via phosphorylation of two translation initiation factors, eIF4E4 and PABP1, which enhance the interaction of eIF4G3 with the m^7^G cap and of PABP1 to poly (A) sequences. It also induces the interaction with the eIF4G3 translation initiation factor to promote global protein translation [72]. CRK3 (orthologue of human CDK1/ *Saccharomyces pombe* cdc2), interacts with different cyclins during the cell cycle (CYC1/CYCA, CYC6, CYC2) [73–75]. CYC6 RNAi in *T. brucei* procyclic forms and inducible deletion of *CRK3* in *L. mexicana* resulted in accumulation of zoids (anucleate cytoplasts) and cells arrested in G2/M [76,77]. CRK2-CYC13 acts as an S-phase CDK by phosphorylating Mcm3 helicase promoting the recruitment of the GINS complex, necessary for the initiation of DNA replication [78]. Another complex CRK12-CYC9 has also been identified in *T. brucei* with RNAi induction of CRK12 in bloodstream parasites resulting in growth arrest with replicative cells presenting enlarged flagellar pocket and defect in endocytosis leading to cell death [79]. Additionally, genetic and chemical approaches were used to show that *Leishmania* CRK12 is essential and a good drug target [80]. Trypanosomatids possess homologues of Tousled-like kinases (TLKs), evolutionary conserved protein kinases involved in development, chromatin assembly, DNA repair, transcription, and chromosome segregation [81]. *T. brucei* TLK1 depletion causes defective spindle formation and chromosome segregation during mitosis [82]. It interacts with the chromosomal passenger complex (CPC), formed by CPC1, CPC2, the kinesin KIN-B and the Aurora B kinase homologue AUK1. AUK1 is regulated both by phosphorylation of two threonine residues in the activation loop of the kinase domain and SUMOylation [83]. Degradation of AUK1 requires two destruction boxes in a mechanism mediated by the E3 ubiquitin ligase APC/C (anaphase-promoting complex/cyclosome) [83]. Two additional aurora kinases have been identified in *T. brucei*; AUK2 depletion led to a growth retardation [6] and sensitises the bloodstream form to genotoxic stress [84], whereas AUK3 is also possibly involved in mitosis and/or cytokinesis [5]. In *L. mexicana* AUK1 and AUK2 are essential for promastigotes, whilst AUK3 is dispensable [2].

## Glossary

### Abbreviations

AGC: group of ePK named after the Protein Kinase A, G, and C families (PKA, PKC, PKG).
Atypical protein kinase (aPK): atypical protein kinases that lack sequence similarity to ePKs, but have been shown experimentally to have protein kinase activity.
Bar-seq: a high-throughput method that quantifies changes in a population by measuring changes in the abundance of mutants containing unique short nucleotide barcodes integrated into the genome.
CAMK: calmodulin/calcium regulated kinase is a ePK group that also includes non-calcium regulated protein kinase families.
CK1: previously known as Casein Kinase 1 group, is now renamed to Cell Kinase 1.
CMGC: ePK group named after the initials of some members (CDK, MAPK, GSK3, and CLK).
Eukaryotic protein kinase (ePK): a superfamily of structurally-related protein kinases to which most protein kinases in eukaryotes belong.
Loss of fitness (LOF): a decrease in the fitness of an organism after gene knockout or gene knockdown.
Mitogen-activated protein kinases (MAPKs): in *Leishmania*, MAPKs have been abbreviated as ‘MPK’, and in *Trypanosoma* as ‘MAPK’.
NEK: protein kinase family named after the never-in-mitososis gene A (NIMA)-related kinase.
Protein kinases: protein kinases are clustered by sequence similarity in the kinase domain and additional information from outer domains, evolutionary conservation and known function, to create groups that are further separated into subfamilies. Typically, two superfamilies, aPK and ePK, have been described with the ePKs further separated into nine groups, including tyrosine kinases and tyrosine kinase-like, AGC, CAMK, CK1, CMGC, STE, RGC, and Other kinases.
Protein serine/threonine kinases: protein kinases that phosphorylate substrate proteins on threonine or serine residues.
Protein tyrosine kinases: protein kinases that phosphorylate substrate proteins on tyrosine residues.
RNA interference target-sequencing (RIT-seq): high-throughput phenotyping using parallel sequencing of RNA interference targets.
Spliced leader (SL): 39 nt capped RNA that is trans-spliced onto every mRNA in trypanosomatids.
STE: ePK group containing homologues of the yeast STE7, STE11, and STE20 genes.

## Highlights

Phosphorylation and the activity of protein kinases are required for successful life cycle transitions in trypanosomatid parasitic protozoa.

Kinome-wide CRISPR and RNAi screens identify protein kinases regulating the cell cycle and life cycle in trypanosomes and *Leishmania*.

Unique features and functions of trypanosomatid protein kinases have been identified and may be exploited as drug targets.

## Outstanding questions

How do we infer functional conservation of signalling pathways in different trypanosomatids?

Which protein kinases are functionally redundant? How do we investigate them, and what are the pathways presenting such redundancy?

Can trypanosomatid protein kinases and their unique features be targeted for the development of a ‘multispecies’ antitrypanosomatid drug?

How do trypanosomatids respond to external signals, and what signalling pathways are involved, for example pH sensing of *Leishmania* in the parasitophorous vacuole?

How are signalling components coordinated into coherent signalling pathways, and are these conserved between species, albeit with different inputs and outputs?
